# Comparison of the Internal and Marginal Adaptation of Implant-Supported Restorations on Titanium Base Using Various Materials: An In Vitro Study

**DOI:** 10.3390/ma18071590

**Published:** 2025-04-01

**Authors:** Oriol Cantó-Navés, Jordi Martí-Vigil, Javier de Medrano, Jiaxing Wu, Oscar Figueras-Alvarez

**Affiliations:** Faculty of Dentistry, Universitat Internacional de Catalunya (UIC), 08017 Barcelona, Spain; oriolcanto@uic.es (O.C.-N.); jdemedrano@uic.es (J.d.M.); od138774@uic.es (J.W.); ofigueras@uic.es (O.F.-A.)

**Keywords:** marginal fit, internal fit, zirconia, doped graphene PMMA, restoration, CAD-CAM, silicone replica technique, SRT

## Abstract

The adaptation of restorations to the titanium base (TiBase) abutments varies depending on the materials and methods used, playing a crucial role in implant and prosthetic success. This in vitro study aims to compare the internal and marginal fit of a titanium interface among three different milled materials: doped graphene PMMA, single-density zirconia, and dual-density zirconia, used for the rehabilitation of CAD-CAM implant-supported single crowns. A digital method based on the silicone replica technique was employed. The silicone reproduction of each fabricated restoration’s inner and basal parts was digitally aligned to the titanium base, measuring the space between them at three predetermined planes: basal, middle, and superior. The material with the worst overall adaptation was dual-density zirconia (0.1 ± 0.03 mm), followed by single-density zirconia (0.09 ± 0.03 mm), and doped graphene PMMA (0.06 ± 0.02 mm). No statistical differences were found in the internal fit, represented by the measurements made at the middle and superior plane, among the materials used (*p* > 0.05). However, the marginal fit of doped graphene PMMA restorations was statistically better than zirconia restorations (*p* < 0.05). No significant differences were observed between the marginal fit of both types of zirconia (*p* > 0.05). Across all three materials, the superior plane showed the best fit, while the marginal plane exhibited the worst.

## 1. Introduction

The introduction of industrially manufactured titanium base (TiBase) abutments, integrated into CAD/CAM libraries, has allowed commercial laboratories to fabricate customized milled abutments or crowns. The main advantage of TiBase abutments is their precise fit, ensuring stable implant connections. Cementing the final prosthesis directly to the TiBase using CAD/CAM blocks with screw access holes permits extraoral bonding, minimizes excess cement, and reduces the risk of cement-related peri-implant bone loss. This approach also optimizes the fit with the implant connection [1,2,3].

Prosthetic design plays a crucial role in the long-term success of implant-supported restorations. One of the key factors influencing peri-implant disease is bacterial plaque accumulation at the abutment–implant interface, which is a major contributor to late-stage implant failure [3,4,5,6].

The basal or marginal gap is the vertical distance between the restoration’s finish line and its cervical margin. This factor is critical for maintaining structural integrity, as marginal discrepancies can expose cement to the oral environment, leading to dissolution and potential microleakage. Over time, this can promote bacterial plaque accumulation [7,8,9]. Although there is no consensus on the acceptable marginal gap, several studies suggest a threshold of 120 μm for successful restorations [7,10,11,12,13,14].

Poor marginal adaptation can significantly compromise the durability of restorations, increasing the risks of plaque accumulation, microleakage, caries, and periodontal or peri-implant disease [10,15,16,17,18,19,20,21]. Similarly, inadequate internal adaptation increases the risk of prosthetic fractures. Ensuring optimal marginal and internal adaptation is essential for implant restorations’ long-term success [7,8,10,15,16,21,22,23,24].

With the widespread adoption of CAD-CAM technology in implant dentistry [11,21], digital methods for assessing marginal and internal fit have become increasingly prevalent. These techniques are used to evaluate inlays, onlays, veneers, crowns, fixed partial dentures, implant abutments, and even full oral restorations. CAD-CAM technology was developed to address three key challenges: ensuring adequate strength for restorations (especially in posterior teeth), achieving high-quality esthetics, and enhancing manufacturing accuracy and efficiency [21,25,26].

Recent computer-assisted technology advancements have refined digital evaluation methods for marginal and internal fit. Various assessment techniques have been proposed [9,12,14,27,28]. One widely used technique is the silicone replica technique (SRT), in which silicone is injected to replicate the internal and marginal adaptation. This technique allows for quantitative analysis after slicing the silicone duplicate [10,14,29,30]. While SRT is cost-effective and non-invasive, it has limitations, including a restricted number of measurable sections and potential material distortion. To improve precision and eliminate manual sectioning, recent studies have integrated digital methodologies with traditional SRTs [14,27,28,31].

Zirconia (Zirconium Dioxide-ZrO_2_) is a high-strength polycrystalline ceramic known for its superior mechanical properties compared to other ceramics. Its natural color and reduced bacterial plaque adhesion have contributed to its widespread use in dentistry [29,30,32,33]. Zirconia can be processed through soft zirconia pre-sintering or hard, fully sintered zirconia milling. Soft zirconia undergoes a 20–30% shrinkage during sintering, a factor that varies depending on the manufacturer [29,30,32].

Polymethyl methacrylate (PMMA) is widely used due to its low density, low elastic modulus, and esthetic qualities [34,35,36]. Graphene, a unique allotropic form of carbon, consists of a single layer of sp^2^ hybridized carbon atoms arranged in a two-dimensional honeycomb structure [35,37,38,39,40]. Recognized as the strongest and thinnest material in the world [25,26,31,41], graphene has been incorporated into PMMA (doped graphene PMMA) [22,23,34,36,38].

Zirconia is one of the leading metal-free materials for fabricating implant-supported restorations [16,24,29,30,32]. In contrast, doped graphene PMMA is a newer material still under research but exhibits promising properties for implant restorations [36,38,39,40]. Both materials are produced through subtractive manufacturing, ensuring high precision in large-scale production [22,32,36]. However, milling has limitations, including reduced precision, tool wear, calibration issues, and challenges in creating complex structures [16,17,21,22,42].

Given the critical role of TiBase adaptation in the success of implant prosthetics, this in vitro study aims to compare the internal and marginal fit of three different CAD-CAM milled materials for implant-supported single restorations: doped graphene PMMA, single-density zirconia, and dual-density zirconia, at the time of manufacturing.

## 2. Materials and Methods

Zirconia and doped graphene PMMA were selected for this comparative study assessing the marginal fit of crowns on titanium implant interfaces (TiBase). The study samples were produced by milling a zirconia disk with a hardness of 1100 MPa and an A3.5 color (BioDynamic, Parma, Italy), a dual-density zirconia disk with 1200/600 MPa hardness and A3.5 color (BioDynamic, Parma, Italy), and a doped graphene PMMA disk (Acrylgraph, Soneja, Spain). Detailed information is provided in Table 1. The crowns were evaluated for their fit to the titanium base (REF: 57657) (Bego Implants, Bremen, Germany).

**Figure 1:**
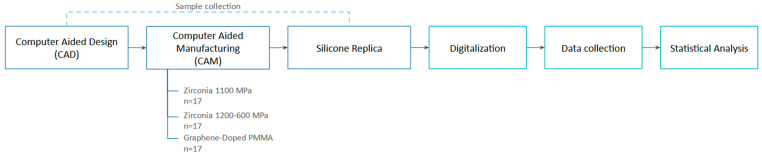
Workflow of the study.

The study measurements were obtained using the tooth-supported digital silicone replica method workflow [28,31]. This method consists of three phases: digitalization of the silicone replica, STL superimposition, and digital measurement (Figure 1).

### 2.1. Computer-Aided Design (CAD)

The sample design was created using Exocad DentalCAD 3.1 Rijeka (exocad GmbH, Darmstadt, Germany). A right maxillary first molar was designed on a TiBase, incorporating the marginal and internal fit parameters specified in the manufacturer’s proprietary dental software library. The design was then exported as a standard tessellation language (STL) file for manufacturing.

### 2.2. Computer-Aided Manufacturing (CAM)

**Figure 2:**
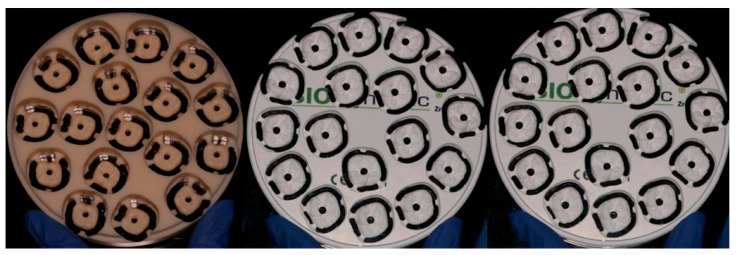
Milled Acrylgraph-doped graphene PMMA disk, BioDynamic 1100 MPa zirconia disk, and 1200/600 MPa disk.

The STL file was imported into the milling machine software DentalCAM (version 8.07.00) to fabricate the samples. A five-axis milling machine (E5 Vhf, vhf camfacture AG, Ammerbuch, Germany) was used, strictly following the manufacturer’s specifications. A total of 51 crowns (17 from each material group) were milled (Figure 2). A new set of milling burs was employed for each material disk to minimize tool wear and ensure precision. The milling process was conducted under controlled temperature conditions in the same laboratory environment.

A statistical power analysis using a one-way ANOVA was conducted based on the reported means and standard deviations. For 90% statistical power (reducing the Type II error risk to 10%), approximately 27 samples (9 samples per group) were required. The study included 51 samples (17 per group), ensuring sufficient statistical power to detect meaningful differences.

Group 1 consisted of doped graphene PMMA crowns. After milling, the individual crowns were separated from the disk using a bur (Diambconflat end FG M 014, Dentsply Sirona, Charlotte, NC, USA). Group 2 included single-density zirconia crowns, while Group 3 comprised dual-density zirconia crowns with a hardness of 600 MPa in the coronal half and 1200 MPa in the basal half. Individual zirconia crowns were separated from the disk following milling using the same bur type. The zirconia samples were sintered in the TABEO-1/M/ZIRKON-100 sintering furnace (MIHM-VOGT GmbH & Co. KG, Stutensee, Germany), following the manufacturer’s specifications (BioDynamic).

### 2.3. Silicone Replica

A silicone replica of each crown’s internal part was created by injecting light-flow addition silicone (Flexitime, Kulzer GmbH, Hanau, Germany) into the TiBase abutment space. The silicone was injected from the occlusal aspect of the crown to the most apical area, ensuring full internal replication. Each crown was then positioned in a heavy body addition silicone base (Flexitime, Kulzer GmbH, Hanau, Germany), and any excess material was trimmed away using a No. 15 scalpel blade (Swann-Morton^®^, Sheffield, UK).

**Figure 3:**
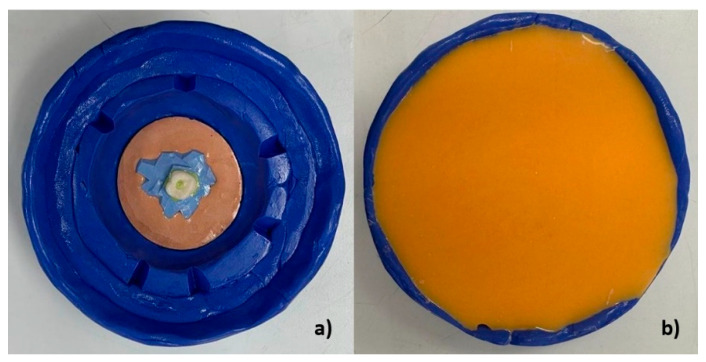
(**a**) Heavy silicone mold with guide crown. (**b**) Custom mold manufacturing with replicating silicone on the heavy silicone mold.

**Figure 4:**
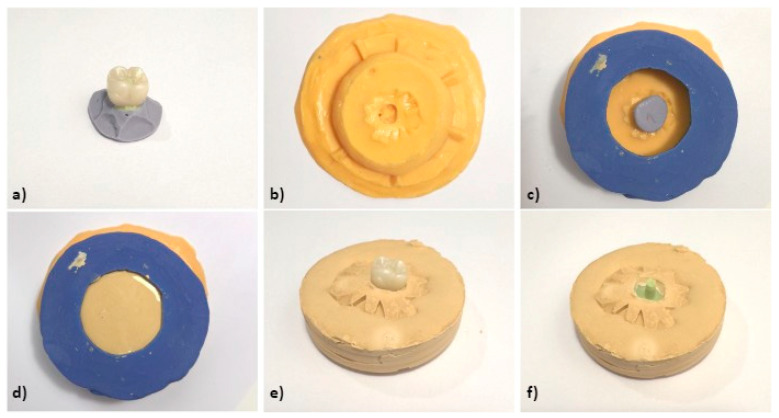
(**a**) Crown with fluid silicone injected inside and heavy silicone base (silicone replica). (**b**) Custom silicone mold for casting. (**c**) Silicone mold with crown and silicone replica. (**d**) Casting process. (**e**) Silicone replica with crown on plaster base. (**f**) Silicone replica on plaster base.

A custom mold was created to ensure that all samples were fabricated in an identical position. A plaster base form was constructed on a previously made silicone replica without removing the crown from the silicone (Figure 3a). This crown with the base form was then placed in a custom silicone mold made from laboratory heavy condensation silicone (Zhermack SpA, Badia Polesine, Italy), allowing for the preparation of a standardized base for all samples (Figure 3 and Figure 4a,b).

A perforated lid was created on the replicating silicone base to facilitate casting all samples in the same base form (Figure 4c–f). This method ensured consistency across samples during scanning, superimposition, and digital measurement. Finally, the TiBase abutment was secured with light addition silicone in one of the crowns, and a silicone replica was produced using the same protocol to maintain the interface position.

### 2.4. Digitization

The 51 silicone replicas and the original TiBase abutment on their plaster bases were scanned using an extraoral scanner (OPTICAL-REVENGE, Open Technologies S.R.l., Rezzato, BS, Italy). The meshes of the silicone replicas and the scanned TiBase were aligned to digitally measure the discrepancy between the original TiBase and the silicone replicas using Limaguide^®^ software (version 1.9.3) (Innovación Dental, S.L., Barcelona, Spain).

### 2.5. Data Collection

The Limaguide^®^ software was used to measure the inner surface of the crowns and the TiBase. Three cutting planes were established: basal, middle, and superior. Four reference points were selected in each plane to ensure consistent measurement across all samples: two in the mesiodistal direction and two in the buccal–lingual direction. Thus, the space between the TiBase and the fabricated crowns was measured at twelve points.

**Figure 5:**
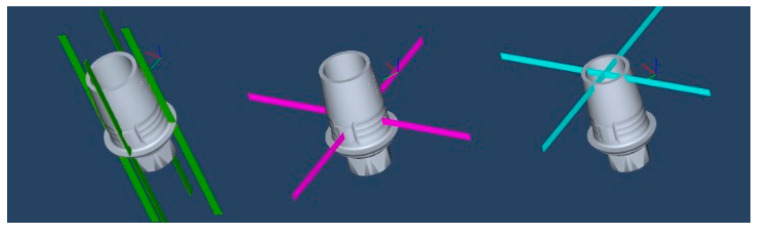
Three predetermined planes: basal (green), middle (purple), and superior (blue).

**Figure 6:**
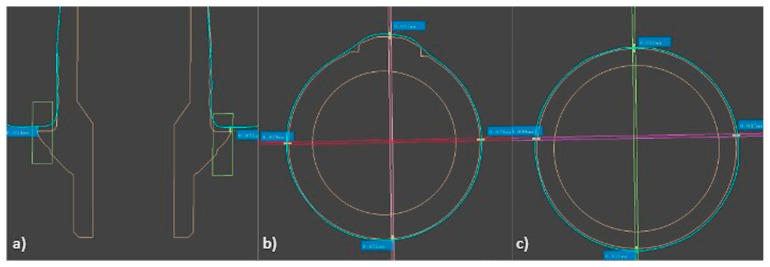
Measurement points of: (**a**) basal plane, (**b**) middle plane, (**c**) superior plane.

Each plane was created using two perpendicular prisms, positioned horizontally to the interface, forming fixed buccal–lingual and mesiodistal planes (Figure 5). The 12 measurement points were labeled from A to L and divided into three groups: basal (A–D), middle (E–H) and superior (I–L). The basal plane represented the marginal fit, while the middle and superior planes represented the internal fit (Figure 6). Vertical measurements were taken at the basal plane for the marginal fit, and horizontal measurements were collected at the middle and superior planes for the internal fit.

The results were obtained by aligning silicone replicas from Groups 1, 2, and 3 with the reference TiBase scan using fixed planes. The space between the inner crown areas and the TiBase was measured in millimeters at four points on each plane across the 51 samples. These measurements were then exported to a Microsoft Excel spreadsheet.

### 2.6. Statistical Analysis

Statistical analysis was performed using SPSS^®^ Statistics 27.0 software (IBM, Armonk, NY, USA). After verifying normality and homoscedasticity using the Shapiro–Wilk and Levene tests, descriptive statistics (means and standard deviations) were computed.

A multifactorial analysis of variance (ANOVA) was conducted with misfit as the dependent variable and two independent factors: material type (doped graphene PMMA, single-density zirconia, and dual-density zirconia) and measurement plane (basal, middle, and superior). A post hoc Least Significant Difference (LSD) test was performed to identify the significant differences between groups. The statistical significance threshold was established at *p* < 0.05 with a 95% confidence interval.

## 3. Results

The mean values and standard deviations for each crown’s measured space, along with the statistical significance levels, are represented in Table 2.

The fit between the titanium base and the 51 crowns fabricated—17 in doped graphene PMMA (Group 1), 17 in single-density zirconia (Group 2), and 17 in dual-density zirconia (Group 3)—was analyzed through multifactorial variance analysis at the three measurements planes: basal, middle, and superior. Both the measurement location (F = 399.930, *p* < 0.001) and the material type (F = 15.500, *p* < 0.001) significantly influenced the measured misfit between the titanium base and the crowns.

**Figure 7:**
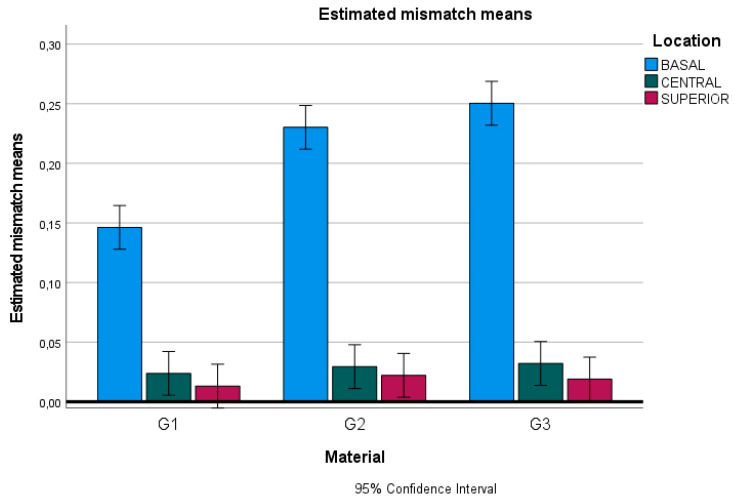
Chart of estimated mismatch means: G1, doped graphene PMMA; G2, zirconia 1100 MPa; and G3, zirconia 600–1200 MPa.

No significant differences were found in the internal fit (middle and superior planes) among the materials used (*p* > 0.05). However, doped graphene PMMA restorations exhibited a statistically inferior marginal fit compared to zirconia restorations (*p* < 0.05). No significant differences were observed between the marginal fit of single-density and dual-density zirconia (*p* > 0.05) (Figure 7).

## 4. Discussion

The success and longevity of indirect restorations are closely linked to their internal and marginal adaptation [10,15]. A poor marginal fit can lead to plaque accumulation, inflammation of adjacent tissues, and an increased risk of periodontal or peri-implant disease [6,15,16,27]. Although the acceptable clinical threshold for the marginal fit varies across studies, many suggest a 120 µm limit, which is lower than the values obtained in this study [10,16,17,18,19,32,33]. Doped graphene PMMA samples demonstrated a near-acceptable marginal fit, while zirconia samples exhibited larger marginal discrepancies, potentially affecting their long-term clinical performance.

Previous studies indicate that the positioning of the samples within the milling disk can influence fabrication accuracy, with centrally positioned samples exhibiting better adaptation than peripheral ones [43]. This factor could explain some of the misfit values observed in this study, as only one disk per material was used. While sample positioning within the disk may introduce minor variations, it does not alter the comparative results between materials, as all samples were milled under the same conditions.

The adaptation of restorations to TiBase abutments plays a crucial role in prosthetic success, as misfit can facilitate bacterial plaque accumulation, leading to gingival inflammation and potential peri-implant bone loss due to occlusal loading (functional or parafunctional) [5,6].

In this in vitro study, we aimed to compare the misfit between a Tibase framework and crowns milled from different materials at the time of manufacturing, using only the methodology previously described in the literature. Doped graphene PMMA, single-density zirconia, and dual-density zirconia were analyzed using the digital silicone replica and digital measurement techniques.

Significant differences in adaptation were observed across measurement planes in all groups. The basal plane (marginal fit) exhibited the poorest adaptation, whereas the middle and superior planes showed better fit across all materials. The middle and superior planes represent the space allowed for the cementing agent, which is pre-set in the proprietary CAD software (Exocad DentalCAD 3.1 Rijeka) parameters.

Doped graphene PMMA restorations exhibited significantly better marginal fit than zirconia restorations. This could be attributed to the material’s lower hardness, which enhances milling accuracy [42,44], the shrinkage effect during zirconia sintering [29,32,44], or the effect of tool wear on harder materials. However, in this study, tool wear effects were minimized by using a new set of burs for each milled disk.

Additional tests, including thermal aging and extraction retention assessments, may impact the basal fit over time. Future studies could employ methodologies such as X-ray diffraction (XRD), scanning electron microscopy (SEM), field emission scanning electron microscopy (FESEM), finite element analysis (FEA), optical microscopy, or stereomicroscopy to observe potential long-term changes.

Several studies have investigated the internal and external adaptations using different methodologies. Refaie et al. compared the adaptation of milled monolithic zirconia and 3D-printed crowns using the silicon replica technique and a microscope at 50× magnification. Their study found that milled zirconia crowns had a mean misfit of 60 ± 20 μm, which was smaller than the values observed in our study [16]. However, their study included printed restorations, while ours focused exclusively on milled materials.

Using the triple-scan protocol, Cantó-Navés et al. evaluated the internal and marginal adaptation of onlay restorations fabricated via subtractive and additive manufacturing methods. Their findings revealed a fit range of 160.57 ± 67.72 to 225.73 ± 11.31 μm, which aligns with the misfit values observed in our study for doped graphene PMMA and zirconia restorations [17].

Park et al. compared the marginal and internal discrepancies of provisional implant restorations fabricated by additive, subtractive, and conventional thermoplastic resin techniques. They found that milled restorations exhibited a total discrepancy of 109.59 μm and a marginal fit of 58.02 μm, ranking second in adaptation after additively manufactured crowns [22]. Our study reported larger marginal discrepancies, particularly in zirconia samples.

Goujat et al. conducted a systematic review of in vitro studies evaluating CAD/CAM inlay and onlay restorations. Most studies reported acceptable fit below 120 μm, highlighting the significant influence of the material used on restoration adaptation [18]. Similarly, our study confirms that material selection plays a critical role in the marginal fit, with doped graphene PMMA outperforming zirconia in this aspect. However, systematic reviews aggregate data from multiple methodologies, which may impact comparability.

El-Farag et al. used a 3D finite element analysis and in vitro testing to assess the impact of CAD/CAM materials on internal and marginal adaptation, fracture resistance, and failure probability of endocrown restorations. Their study, which used the silicone replica technique and a stereomicroscope at ×25 magnification, found that zirconia restorations exhibited the highest marginal discrepancies, while PEEK restorations showed the lowest [23]. These findings align with our results, as zirconia restorations in our study demonstrated greater marginal misfit compared to doped graphene PMMA.

Ferrini et al. analyzed the marginal precision in CAD/CAM crowns fabricated from zirconia, lithium disilicate, and composite over cobalt-chromium dies, using microphotographs at 580× magnification. They reported significant variations between the materials, with zirconia showing the best fit, followed by composite, and lithium disilicate. Their mean values for zirconia restorations (21.45 ± 12.58 μm) were considerably lower than those observed in our study [19]. These discrepancies may be due to the differences in scanning and milling precision or the methodologies used in each study.

One limitation of this study is the use of a single disk for each material, which may introduce variability in the milling process. However, since all samples were fabricated under identical conditions and positioned the same way within the disk, this limitation does not undermine the comparative validity of our results.

Additionally, this study did not incorporate thermal aging or mechanical testing. Future research should include mechanical strength evaluations, thermal cycling, and retention for assessments, particularly for doped graphene PMMA, which remains a promising but less-explored material. Moreover, alternative evaluation methodologies such as XRD, SEM, FESEM, FEA, optical microscopy, and stereomicroscopy could provide additional insights into material behavior over time. These techniques should be considered in future investigations to explore long-term adaptation and clinical performance further.

## 5. Conclusions

Within the limits of this study, which evaluated the misfit at the time of manufacturing, the following conclusions can be drawn:

1. Doped graphene PMMA restorations demonstrated better marginal adaptation to TiBase compared to both single-density and dual-density zirconia.
2. No statistically significant differences were found in the internal adaptation of restorations fabricated from doped graphene PMMA, single-density zirconia, and dual-density zirconia.
3. Among the three tested materials, the marginal plane exhibited the highest degree of misfit, whereas the superior plane demonstrated the best adaptation.
4. The digital silicone replica technique combined with a digital measurement proved to be a reliable method for assessing the fit of different CAD-CAM milled materials in implant-supported restorations.
5. Material selection significantly influences marginal adaptation, with doped graphene PMMA achieving superior marginal fit at the time of manufacturing compared to zirconia.

Although doped graphene PMMA demonstrated a better marginal fit, this study does not assess its long-term clinical performance or mechanical properties. Further in vitro research, including thermal aging, mechanical strength testing, pull-out retention analysis, bacterial adhesion assessments, and SEM/TEM imaging, are required to validate these findings and evaluate their implications for prosthetic success.

## Figures and Tables

**Table 1:** Materials used in this study, their product name, manufacturer, location, and manufacturing method.

Material	Product Name	Manufacturer	Location	Manufacturing Method
Zirconia 1100 MPa	BioDynamic Zr	Biodynamic	Parma, Italy	Milling + Sintering
Zirconia 1200-600 MPa	BioDynamic Zr	Biodynamic	Parma, Italy	Milling + Sintering
Doped graphene PMMA	Acrylgraph	KPT59 S.L.	Soneja, Spain	Milling

**Table 2:** Means ± standard deviations of the crown fit from group 1, group 2, and group 3 in the three predetermined planes, and *p*-value according to ANOVA. Superscript letters indicate significantly different groups at *p* < 0.05. * indicate significant differences at *p* < 0.05.

	Basal Plane	Middle Plane	Superior Plane
Doped graphene PMMA (G1)	0.1463 ± 0.4025 ^a^	0.0238 ± 0.00669	0.0132 ± 0.00446
Zirconia 1100 MPa (G2)	0.2303 ± 0.7744 ^b^	0.0295 ± 0.00896	0.0222 ± 0.00819
Zirconia 1200-600 MPa (G3)	0.2505 ± 0.7268 ^b^	0.0321 ± 0.00741	0.0190 ± 0.00767
	<0.05 *	>0.05	>0.05

## Data Availability

The original contributions presented in this study are included in the article. Further inquiries can be directed to the corresponding author.

## Abbreviations

The following abbreviations are used in this manuscript:

SRT	Silicone replica technique
PMMA	Polymethyl methacrylate
STL	Standard tessellation language
CAD	Computer-aided design
CAM	Computer-aided manufacturing
